# Genome-Wide Identification, Characterization, and Expression Analysis of *VQ* Gene Family in *Salix suchowensis* Under Abiotic Stresses and Hormone Treatments

**DOI:** 10.3390/plants14101431

**Published:** 2025-05-10

**Authors:** Hongjuan Wang, Yujiao Wang, Yongle Wang, Jiabao Zhu, Lei Chen, Xiaoming Yan, Chun Yu, Benli Jiang

**Affiliations:** 1 Institute of Industrial Crops, Anhui Academy of Agricultural Sciences, Hefei 230001, China; 2 College of Agriculture, Anhui Science and Technology University, Fengyang 233100, China

**Keywords:** *Salix suchowensis*, valine glutamine (VQ), gene family, abiotic stress, hormone

## Abstract

The valine glutamine (VQ) proteins are transcription cofactors involved in various aspects of plant biology, including growth, development, and stress resistance, making them an attractive target for genetic engineering aimed at enhancing plant resilience and productivity. However, comprehensive reports or systematic studies on VQ cofactors in *Salix suchowensis* remain lacking. In this study, we analyzed *SsVQ* genes using bioinformatics methods based on the *Salix suchowensis* genome database. Expression profiles were further investigated through qRT-PCR under six treatments: PEG, NaCl, 40 °C, ABA, SA, and MeJA. A total of 39 *SsVQ* genes were identified, with phylogenetic analysis classifying them into seven groups. Collinearity analysis suggested that *SsVQ* gene amplification primarily resulted from whole genome duplication (WGD) or segmental duplication events. Ka/Ks ratios indicated that willow *VQ* genes have undergone predominantly purifying selection. Gene structure analysis revealed that *SsVQ* genes are intronless. Multiple sequence alignment showed that SsVQ19 shares similarity with PtVQ27, containing a hydrophilic threonine (T) residue preceding the VQ amino acid residues. Furthermore, genes within each group exhibited conserved structures and VQ motifs. Promoter and expression analyses suggested the potential roles of *SsVQ* genes in regulating willow responses to environmental stresses and hormonal signals. Most *SsVQ* genes displayed differential expression at specific time points, with six members (*SsVQ2*, *SsVQ9*, *SsVQ12*, *SsVQ23*, *SsVQ32*, and *SsVQ34*) showing sustained high-amplitude expression profiles across treatments. Notably, *SsVQ34* demonstrated pronounced transcriptional induction under PEG stress, with expression levels upregulated by 62.29-fold (1 h), 49.21-fold (6 h), 99.9-fold (12 h), and 201.50-fold (24 h). Certain *SsVQ* genes showed co-expression under abiotic/hormonal stresses, implying synergistic functions. Paralogous gene pairs exhibited stronger co-expression than non-paralogous pairs. This study provides novel insights into the structural and functional characteristics of the *VQ* gene family in *Salix suchowensis*, establishing a foundation for future research on the stress-resistance mechanisms of willow *VQ* genes.

## 1. Introduction

*Salix suchowensis* is a shrub willow species that is native to China [1,2]. This species is dioecious [3]; it has the characteristics of small individual size [4], rapid maturation [4], and easy reproduction [5]. Its flexible and uniform branches make it an ideal material for willow weaving, while its products are safe, environmentally friendly, and durable. Notably, *S. suchowensis* is not only a fast-growing economic tree but also an ecologically valuable species [6]. With strong tolerance to waterlogging, it grows well on riverbanks, making it an excellent tree species for soil and water conservation and embankment consolidation [7]. The individual size and short juvenile phase facilitate large-scale field experiments, and there are abundant natural variations, making it an ideal material for genetic research [2]. The recent publication of its full genome sequence further enables molecular genetic studies [2].

Transcription cofactors interact with transcription factor proteins to form complexes, achieving precise and effective regulation of target genes. Research has shown that transcription cofactors can affect DNA-binding ability, transcriptional activation or inhibition activity, subcellular localization, protein stability, and so on [8]. The VQ protein is a type of transcriptional cofactor, widely present in plants, named after its highly conserved VQ motif (FxxhVQxhTG), where x is any amino acid residue and h is a hydrophobic amino acid residue. VQ motifs can be categorized based on differences in the last three amino acids (hTG) [9,10]. For example, *Arabidopsis thaliana* (Arabidopsis) VQ motifs include six combinations (LTG, FTG, VTG, YTG, LTS and LTD), whereas *Oryza sativa* (rice) and *Populus trichocarpa* (poplar) have four (LTG, FTG, VTG, and ITG) and three (LTG, FTG, and VTG), respectively [11,12,13]. The amino acid sequences outside the VQ motif exhibit diversity, which is consistent with the functional diversity of the VQ protein family [14]. Additionally, most *VQ* genes lack introns and encode relatively short proteins (<300 amino acids) [15].

*VQ* genes are involved in plant growth, development, and stress responses [16]. For instance, *AtVQ29* functions as a transcriptional repressor, inhibiting hypocotyl elongation in response to light and potentially enhancing the transcriptional activity of *PIF1* during early seedling development [17]. *BoVQ25-1* plays an important role in pollen germination [18]. *AtVQ18* and *AtVQ26* act as antagonists of *ABI5* to maintain precise levels of ABA signaling, thereby finely regulating seed germination and seedling establishment [19]. *AtVQ12* and *AtVQ29* negatively regulate plant basal resistance against *Botrytis cinerea* [20]. *TaVQ4-D* overexpression in Arabidopsis and wheat enhances drought tolerance, whereas its silencing in wheat reduces drought tolerance [21]. The Arabidopsis lines overexpressing *PeVQ28* enhanced resistance to salt stress and sensitivity to ABA, along with the upregulation of salt- and ABA-responsive genes [22].

In previous studies, VQ proteins primarily interact with WRKY transcription factors to regulate physiological processes. *AtVQ10* physically interacts with *AtWRKY8*, positively regulating immunity against *B. cinerea* [23]. *AtVQ20*, expressed specifically in pollen, interacts with *AtWRKY2/34* to control pollen development [24]. *SIBs* inhibit *WRKY75* function, negatively modulating ABA-mediated leaf senescence and seed germination [25]. A regulatory module, *OsPUB73-OsVQ25-OsWRKY53*, finetunes rice broad-spectrum disease resistance and growth at transcriptional and posttranslational levels [26]. *TaVQ25-A* functions as a positive regulator of ABA-related leaf senescence by interacting with *TaWRKY133* in vitro and in vivo [27]. *MdVQ10* promotes wound-triggered leaf senescence in association with *MdWRKY75* and undergoes antagonistic modulation of *MdCML15/MdJAZs* in apple [28]. In tomato, *SlWRKY37* positively regulates JA and dark-induced leaf senescence and interacts with *SlVQ7* to improve its own stability [29]. In shoots, *AtVQ10* activation of cell division was counteracted by *AtWRKY33*-exerted repression, thus leading to a dwarf bushy phenotype in plants with enhanced *AtVQ10* expression in a *wrky33* knock-out background [30]. Although direct VQ-WRKY interaction evidence in *S. suchowensis* is lacking, studies in its close relative *S. psammophila* revealed that *SpWRKY33* overexpression in Arabidopsis significantly upregulated a *VQ* gene, implicating its potential role in *SpWRKY33*-mediated drought tolerance [31].

To date, the *VQ* gene family has been genome-wide identified in plants such as Arabidopsis [11], rice [12,32], poplar [13], grape [33], and bamboo [34]. However, no comprehensive identification or expression profile analysis of the *VQ* gene family has been reported in *S. suchowensis*. *S. suchowensis* exhibits strong environmental resilience, and studying its VQ gene family may reveal novel stress-tolerance mechanisms applicable to plant improvement. In this study, we identified 39 *SsVQ* genes in the *S. suchowensis* genome and analyzed their sequence characteristics, protein structures, and phylogenetic relationships. Additionally, we selected 13 paralogous pairs (26 genes) from each phylogenetic group for qRT-PCR analysis to investigate their responses to three abiotic stresses (PEG, NaCl, and 40 °C) and three stress-related hormones (ABA, SA, and MeJA). Our results demonstrated that all 26 *SsVQ* genes were stress-responsive, providing a foundation for understanding the functional mechanisms of VQ proteins in plant stress resistance and offering potential genetic resources for improving stress-tolerant cultivars.

## 2. Results

### 2.1. Identification of the SsVQ Genes and Analysis of Physicochemical Properties

**Figure 1:**
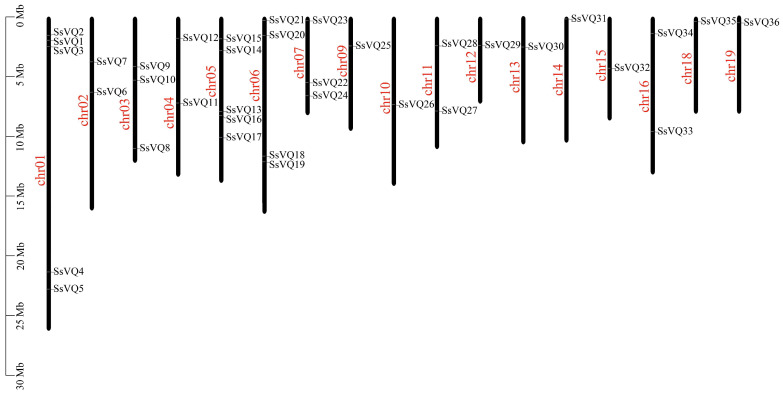
Chromosomal location of *VQ* genes in *S. suchowensis*.

A total of 39 *VQ* family members were identified in the *S. suchowensis* genome. These genes were renamed *SsVQ1* to *SsVQ39* based on the information of chromosomal localization and sequenced ID (Table 1). Three genes (*SsVQ37*, *SsVQ38*, and *SsVQ39*) were located on unassembled scaffolds, while the remaining 36 *SsVQ* genes were unevenly distributed across 17 of the 19 chromosomes (Figure 1). Chromosomes chr8 and chr17 lacked *VQ* genes, whereas chr1 and chr5 contained the highest number of *SsVQ* genes (five genes each). Physicochemical analysis revealed that SsVQ proteins ranged in length from 105 to 497 amino acids (aa), with 31 (79.49%) being shorter than 300 aa and 8 (20.51%) exceeding this length. Their molecular weights varied between 11,418.14 Da and 53,367.44 Da, with predicted isoelectric points (pI) ranging from 4.08 to 10.43. Subcellular localization predictions (Wolf PSORT) indicated that 29 SsVQ proteins were nuclear, 1 was localized to the plasma membrane, 3 to the chloroplast, 1 to the mitochondrion, and 5 to cytosol (Table 1).

### 2.2. Phylogenetic Analysis of VQ Genes

**Figure 2:**
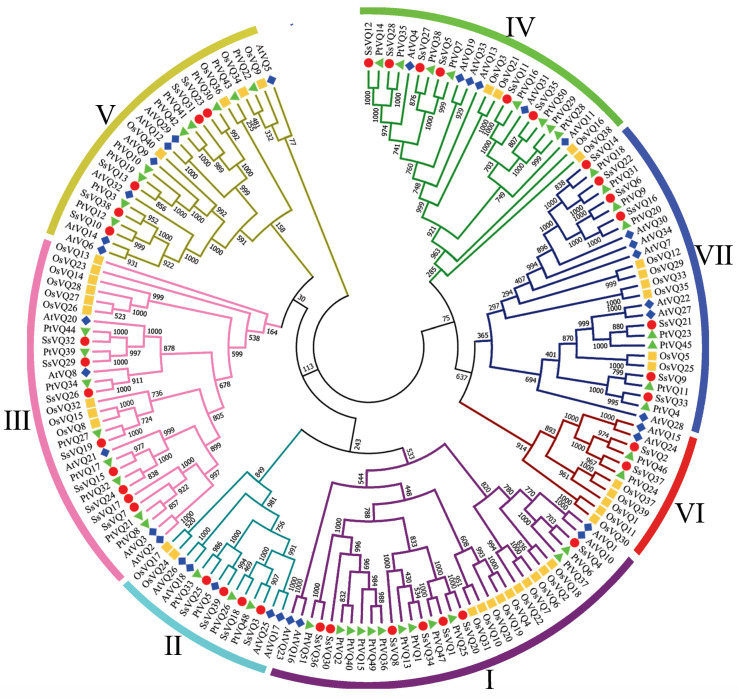
Phylogenetic analysis of *VQ* genes from *S. suchowensis*, Arabidopsis, poplar, and rice. Willow, Arabidopsis, poplar, and rice are denoted by red, blue, green, and yellow shapes, respectively. Numbers I–VII indicate different groups, and the different colors in the outermost circle represent different groups.

Multiple sequence alignment was performed using the full-length VQ protein sequences of willow, Arabidopsis, rice, and poplar, and a phylogenetic tree (NJ tree) was constructed to analyze the evolutionary relationships of VQ family members (Figure 2). According to the results of phylogenetic analysis, the *S. suchowensis* VQ family members were divided into seven groups. Notably, the number of VQ members in each group showed limited divergence between *S. suchowensis* and *A. thaliana*, with Groups II, IV, and VI containing identical gene counts. Strikingly, the NJ tree demonstrated that 84.6% of *S. suchowensis VQ* genes (33 *SsVQ* genes) formed sister pairs with poplar homologs (e.g., *SsVQ12*-*PtVQ14*), indicating their origin from common ancestry.

### 2.3. Duplication Events of VQ Genes

**Figure 3:**
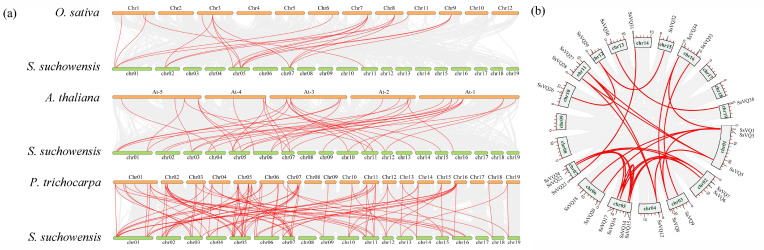
Collinearity analysis. (**a**) *VQ* gene collinearity between willow and other species. (**b**) Collinearity analysis of *VQ* gene in *S. suchowensis*.

To understand the evolutionary mechanism of the *VQ* gene family in *S. suchowensis*, the duplication events of the 36 *SsVQ* genes were analyzed. According to the results of multi-species collinearity analysis, *S. suchowensis VQ* gene had collinearity with all the three plants, while it has the least collinearity pairs with rice (20 pairs, accounting for 25.0% of all *SsVQ* genes), followed by Arabidopsis (40 pairs, accounting for 66.7%), and the most collinearity pairs with poplar (101 pairs, accounting for 100.0%), respectively (Figure 3a). The findings indicated that dicotyledonous plants exhibited a stronger conservation of the *VQ* gene, and there was a very close genetic relationship between willow and poplar. *S. suchowensis* intraspecific collinearity analysis revealed 28 collinearity pairs among 27 *SsVQ* genes (accounting for 75.0%) (Figure 3b). Both intraspecific and interspecific collinear pairs were attributed to whole genome duplication (WGD) or segmental duplication events, indicating that these mechanisms predominantly drove the expansion of the *SsVQ* gene family.

To evaluate the impact of selective pressure on the expansion of the *VQ* gene family, we calculated the Ka/Ks ratios of the *SsVQ* genes for collinearity pairs in four different species. As shown in Table S1, all of the Ka/Ks ratios were found to be less than 1, with a range from 0.10 to 0.62, indicating that willow *VQ* genes had primarily undergone purifying selection.

### 2.4. The Structure and Motif Analysis of the SsVQ Genes

**Figure 4:**
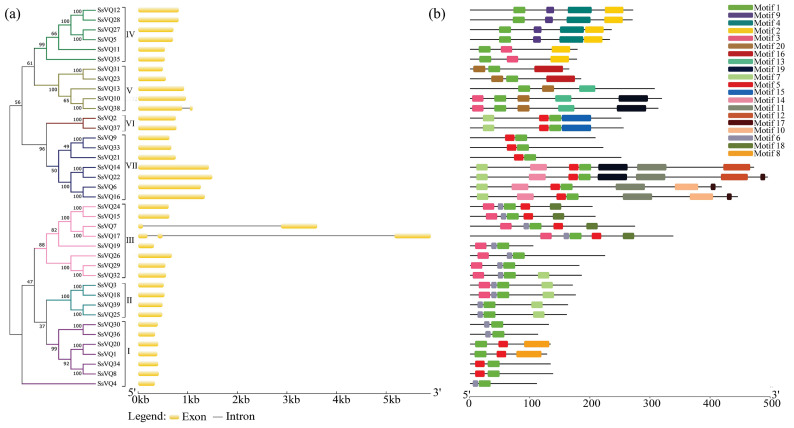
The structures and motifs of the *SsVQ* genes in *S. suchowensis*. Numbers I–VII indicate different groups. (**a**) The gene structures of the *VQ* gene family are shown. (**b**) Protein motifs in SsVQ members are represented by colorful boxes, each denoting a distinct motif.

In order to gain insights into the structural diversity, we conducted an analysis of the Exon/intron structure of each willow *VQ* gene. The results revealed that the majority of *SsVQ* genes did not contain introns, while only *SsVQ7*, *SsVQ17,* and *SsVQ38* were found to contain one or two introns (Figure 4a). To obtain a better analysis of the structural feature of the 39 SsVQ proteins, the conserved motifs were predicted by the MEME program (Figure 4b). A total of 20 different conserved motifs were identified, and each motif was annotated using the Pfam and SMART websites. The length and conserved amino acid sequences of the 20 motifs are shown in Table S2. The results showed that motif 1 was a VQ motif (PF05678), and motif 2 was an RNA recognition motif (PF00076), both of which had mRNA binding function, while the other motifs had no functional annotation. All SsVQ proteins contained motif 1, while only group IV genes contained motif 2. In addition, SsVQ proteins within the same group were composed of similar motifs, while those in different groups showed more differences in motif composition.

**Figure 5:**
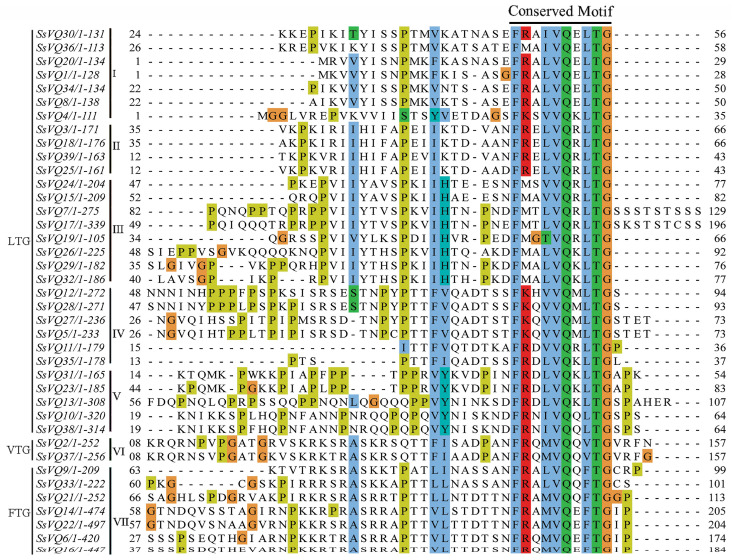
Multiple sequence alignment of the VQ domain of 39 SsVQs, with conserved amino acids shaded in different colors. The color shaded areas indicate several conserved residues.

In order to better understand the similarities and differences of SsVQ motifs, multiple sequence alignments were performed (Figure 5). The typical amino acid sequence of the VQ motif is FxxhVQxhTG, where h is a hydrophobic amino acid residue. All VQ motifs of willow, except SsVQ19, were found to be FxxhVQxhTG. However, SsVQ19 contained a hydrophilic (T: Threonine) rather than a hydrophobic amino acid before the VQ amino acids. According to the classification of the last three amino acids (hTG), the 39 SsVQ proteins contained three VQ motif types: FxxxVQxLTG, FxxxVQxFTG, and FxxxVQ/HxVTG. These motifs are distributed as follows: 30 in group I–V, 7 in group VII, and 2 in group VI. The motif types of SsVQs and PtVQs were consistent. However, SsVQ proteins lacked the FxxxVQxLTD/S, FxxxVQxYTG, and FxxxVQxITG motifs in contrast to AtVQs and OsVQs [6,7].

### 2.5. Identification of Cis-Elements in the Promoters of SsVQ Genes

**Figure 6:**
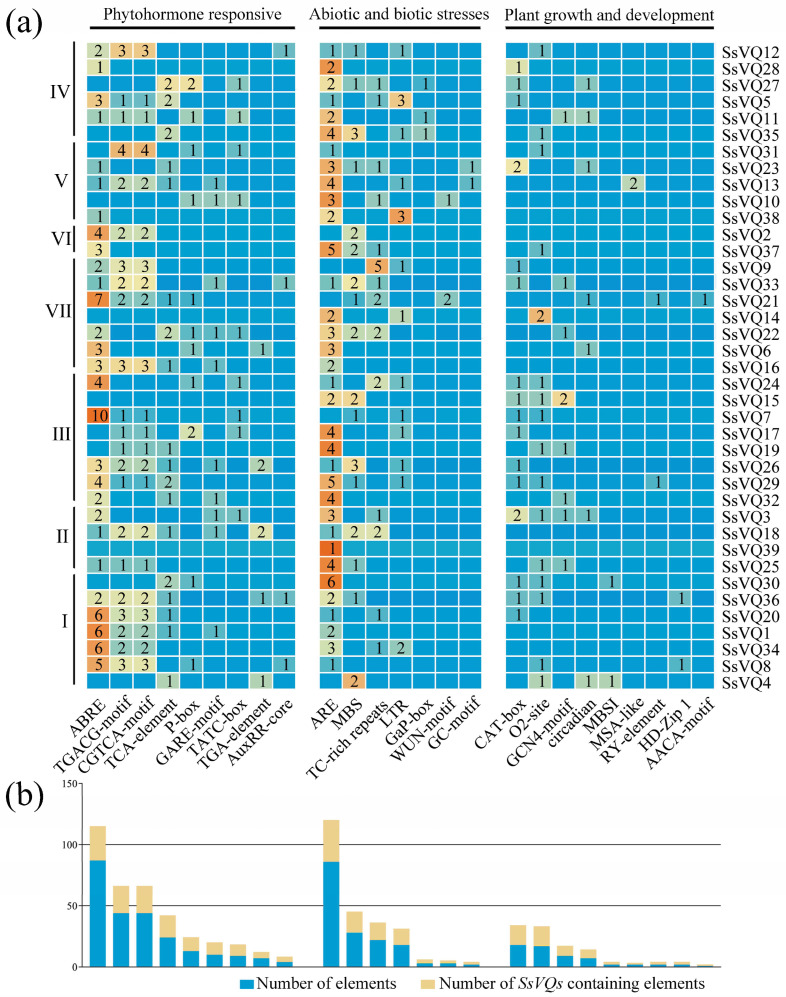
Cis-acting element analysis of the *SsVQ* gene family. Numbers I–VII indicate different groups, numerals 1–10 denote the count of cis-elements, and color gradients reflect quantitative variations. (**a**) Number of each cis element upstream of each *SsVQ* gene. (**b**) The total number of statistics of cis elements and *SsVQs*.

To analyze the regulatory mechanism of the *SsVQ* gene expression, the putative cis-acting elements in the 2000 bp DNA sequences upstream of the start codon were identified (Figure 6). In all, the promoter region analysis revealed the presence of 242 phytohormone response elements, 162 stress-related response elements, and 60 growth and development elements.

In the category of hormone-responsive genes, a total of 28 *SsVQ* genes were identified to be involved in the ABA-signaling pathway, containing a total of 87 ABREs (ABA response elements). Additionally, 22 *SsVQs* were found to be associated with a MeJA response, encompassing 44 TGACG motifs and 44 CGTCA motifs. Furthermore, there were 18 *SsVQs* implicated in SA responsiveness, containing a total of 24 TCA elements. Moreover, 32 gibberellin-response elements and 11 auxin-response elements were found in the promoter region.

In the category of abiotic and biotic stresses, 17 *SsVQs* were found to contain 28 MYB-binding sites involved in drought-inducibility (MBS). Additionally, 14 *SsVQs* contained 22 cis-acting elements involved in defense and stress responsiveness (TC-rich repeats), while 13 *SsVQs* contained 18 cis-acting elements involved in low-temperature responsiveness (LTR). Furthermore, the analysis revealed that 34 *SsVQs* contained a total of 88 anaerobic/anoxic-induced elements (ARE, GC-motif), with only two *SsVQs* containing WUN motifs.

Promoter analysis revealed that the *SsVQ* genes may potentially play an active role in hormone response or stress response, or both. Moreover, it was observed that 31 *SsVQs* contain a total of 60 elements related to plant growth and development.

### 2.6. Expression Patterns of the SsVQ Genes in Response to Abiotic Stress and Exogenous Hormone

Under adverse conditions, plants can cope with the stress and maintain normal growth and development by regulating the expression levels of some genes. In order to investigate the role of paralogous *SsVQ* genes in willow stress regulation, we conducted qRT-PCR analysis on 13 paralogous SsVQ gene pairs under stress- and hormone-treated conditions. Though unselected, the remaining 13 genes may still hold unexplored functions worth further investigation through alternative experimental strategies.

**Figure 7:**
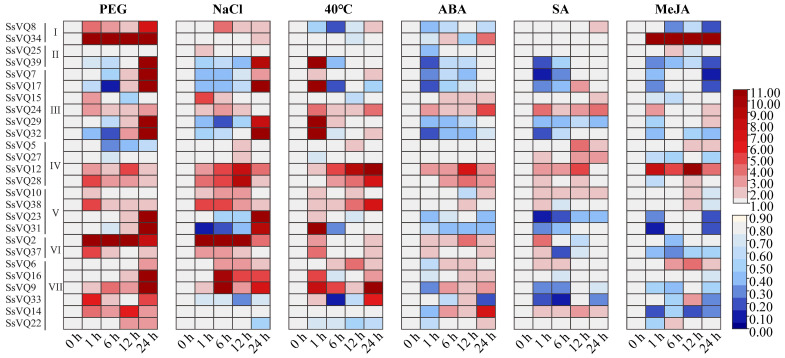
Heat map showing 26 *SsVQ* gene-expression profiles under 3 abiotic stresses and 3 phytohormones based on qRT-PCR. The color scale represented relative expression levels, with red and blue indicating increased or decreased transcript abundance, respectively.

To examine the response of *SsVQ* genes to abiotic stresses, this study employed qRT-PCR to analyze their expression levels under drought, salt, and high-temperature conditions. Overall, most of the *SsVQs* showed increased expression levels in response to abiotic stress conditions (Figure 7 and Figure S1). When using PEG to simulate drought stress, there were significant changes in the expression levels of 24 *SsVQ* genes, except for *SsVQ25* and *SsVQ27*. Notably, the expression levels of 88.5% (23/26) of *SsVQ* genes were significantly upregulated at varying time points. Specifically, at 1 h, 6 h, 12 h, and 24 h, the expression levels of *SsVQ2* and *SsVQ34* were upregulated by 35.46 and 62.29 times, 11.36 and 49.21 times, 13.25 and 99.9 times, and 8.11 and 201.50 times, respectively. On the other hand, 60.9% (14/23) of *SsVQ* genes (*SsVQ6*, *7*, *8*, *9*, *16*, *17*, *22*, *23*, *24*, *29*, *31*, *32*, *34*, and *39*) peaked at 24 h, with *SsVQ7*, *9*, *16*, *17*, *23*, *29*, *31*, *32*, *34*, and *39* being more than 11-fold different. In addition, *SsVQ17* was significantly downregulated to 0.14 at 6 h. After NaCl treatment, significant changes were observed in the expression levels of 24 out of 26 *SsVQ* genes, with the exception of *SsVQ5* and *SsVQ14*. The expression levels of 84.6% (22/26) of *SsVQ* genes were significantly upregulated at different time points, with *SsVQ2* reaching 17.07, 15.94, 12.18, and 3.76 times at 1 h, 6 h, 12 h, and 24 h, respectively. Additionally, the *SsVQ31* gene was significantly downregulated to 0.12 at 1 h. Under high-temperature stress at 40 °C, the expression levels of 84.6% (22/26) of *SsVQ* genes changed significantly, except *SsVQ10*, 25, 27, and 34. Particularly, 73.1% (19/26) of *SsVQ* genes had significantly upregulated expression levels at various time points. *SsVQ17*, *29*, *31*, *32*, and *39* were promptly upregulated by more than 10-fold at 1 h, followed by a dramatic decline. Additionally, *SsVQ17* and *SsVQ33* showed significant downregulation more than 5-fold (<0.2) at 6 h.

Furthermore, a comprehensive analysis of *SsVQ* gene expression was conducted following treatments with three exogenous phytohormones (ABA, SA, and MeJA), respectively. The results indicated that more *SsVQ* genes were downregulated under hormone stress than abiotic stress (Figure 7 and Figure S1). Under ABA stress conditions, significant changes in the expression levels of 88.5% (23/26) of *SsVQ* genes were observed, with the exception of *SsVQ5*, *SsVQ15*, and *SsVQ23*. Further, 39.1% (9/23) of *SsVQ* genes exhibited a significant decrease at various time points, with six *SsVQ* genes showing the most pronounced decline at 1 h. In contrast, 65.2% (15/23) of *SsVQ* genes showed a significant increase, with 14 reaching their peak expression at 12 h or 24 h. After SA treatment, except for *SsVQ16*, *SsVQ22*, *SsVQ25*, and *SsVQ34*, there were significant changes in the expression levels of 84.6% (22/26) of *SsVQ* genes. At various time points, the expression levels of 45.5% (10/22) of *SsVQ* genes exhibited a significant decrease, with seven *SsVQ* genes showing the most decrease at 1 h. Additionally, 63.6% (14/22) of *SsVQ* genes demonstrated a significant increase in expression levels. However, all upregulated expression levels were less than 5-fold. Under MeJA treatment, except *SsVQ10* and *SsVQ29*, the expression level of 92.3% (24/26) of *SsVQ* genes was significantly changed. Further, 61.5% (16/26) of *SsVQ* genes were downregulated, and the expression levels of *SsVQ14* and *SsVQ31* were the lowest and downregulated by more than 5-fold at 1 h of treatment. Among them, the expression levels of *SsVQ7*, *SsVQ8*, *SsVQ23*, and *SsVQ39* were the lowest and downregulated by more than 5-fold after treatment for 24 h. In contrast, 50% (12/24) of *SsVQ* genes demonstrated a significant increase, with eight reaching their peak expression at 12 h. In addition, the expression levels of *SsVQ34* were significantly upregulated in all treatment periods, and the upregulation multiples ranged from 16.35 to 63.50. In particular, *SsVQ12* was significantly upregulated under all treatments, with peak values of 4.96 under PEG, 8.34 under NaCl, 12.08 under 40 °C, 7.42 under ABA, 4.61 under SA, and 12.80 under MeJA.

### 2.7. Co-Expression Networks of the SsVQ Genes

**Figure 8:**
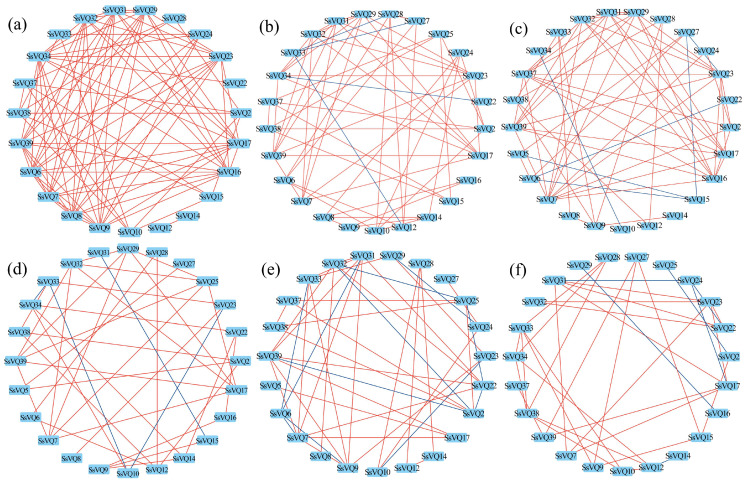
Correlation analysis of *SsVQ* gene family based on the Pearson correlation coefficients (PCCs) of relative expression data between gene pairs. Panels (**a**–**f**) represent the co-expression networks of *SsVQ* genes under PEG, NaCl, 40 °C, ABA, SA, and MeJA treatments. Red or blue lines are drawn to indicate a PCC ≥ 0.8 or ≤−0.8, with *p*-value ≤ 0.05.

The expression patterns of *SsVQ* genes showed various changes under abiotic stresses and hormonal treatments. In order to visualize the correlation between the changes of *SsVQ* gene expression, we drew co-expression networks based on Pearson correlation coefficients (PCC) between expression patterns. As illustrated in Figure 8, the number of *SsVQ* gene co-expressions was highest under PEG treatment, and all of them exhibited positive correlations. In contrast, the co-expression relationships of *SsVQ* genes varied under other treatments, with most showing a positive correlation and a few showing a negative correlation. The order of co-expression numbers from highest to lowest was PEG > 40 °C > NaCl > SA > ABA > MeJA. Additionally, the homologous pair *SsVQ7* and *SsVQ17* showed a consistent positive correlation across all treatments.

## 3. Discussion

VQ proteins are non-plant-specific proteins, but they are also present in some fungi, lower animals, and bacteria [35]. Previous studies have highlighted the significant role of VQ proteins in various aspects of plant biology, including growth, development, and stress responses [16,17,18,19,20,21,22]. While our knowledge of the *VQ* genes in *S. suchowensis* is limited, further research is necessary to fully understand their functions in this particular species. Therefore, we utilized bioinformatic methods to perform a genome-wide analysis of *VQ* genes in *S. suchowensis* and investigate their expression patterns under various stresses and hormone treatments.

This study identified 39 SsVQ proteins in *S. suchowensis*, all of which contain a conserved FxxxVQxxTG motif. In comparison, *P. trichocarpa*, another member of the Salicaceae family, contains 51 PtVQ proteins. These findings confirm that poplar retained more “Salicoid” duplicates than willow after their divergence [2]. The uneven distribution of 39 *SsVQ* genes on 17 of the 19 chromosomes, except for chromosomes 8 and 17, is consistent with the distribution pattern observed in poplar. Subcellular localization prediction showed that 29 SsVQ proteins were located in the nucleus, while 10 SsVQ proteins were predicted to be located in the chloroplast, mitochondrion, plasma membrane, or cytosol, indicating that members of the SsVQ protein family may play roles in different locations.

A total of 39 *SsVQ* genes were divided into seven groups based on a comprehensive phylogenetic analysis conducted among *A. thaliana*, *P. trichocarpa*, *S. suchowensis*, and *O. sativa*. The phylogenetic tree analysis revealed that SsVQs and PtVQs consistently clustered together, likely due to their shared classification within the Salicaceae family. Genes that were closely related within the same group exhibited similar gene structures, characterized by comparable intron numbers and exon lengths. In this study, 92.31% of SsVQ genes (36/39) were intronless, providing further evidence that most *VQ* genes in higher plants are intronless [14]. Some researchers propose that the introns of the *VQ* gene have evolved independently in recent history [36], whereas others contend that these introns were gradually lost throughout evolution as a result of varying selective pressures [15,37,38]. Most SsVQ proteins (79.49%) comprised less than 300 amino acids, which is similar to the study in Arabidopsis, rice, poplar, maize, coix, tobacco, and *Brassica juncea* [11,12,13,15,36,37,38].

Within each group, both exon/intron structures and motif compositions exhibited a high degree of conservation. The primary VQ motif identified in group VI across willow, Arabidopsis, rice, and poplar is FxxxVQxVTG, while FxxxVQxFTG is predominantly observed in group VII. These variations in conserved amino acid sequences may contribute to functional differentiation among the groups. Multiple sequence comparisons revealed that all VQ motif amino acid sequences in willow are characterized by the pattern FxxhVQxhTG, with the exception of SsVQ19. SsVQ19 found in willow and PtVQ27 found in poplar are Orthologous genes. Notably, SsVQ19 showed a similarity to PtVQ27 with a hydrophilic amino acid residue (threonine, T) preceding the VQ amino acid residue. Furthermore, no such conserved motif variation was observed in Arabidopsis thaliana or rice [11,12].

The analysis of upstream promoter sequences for *SsVQ* genes revealed the presence of several cis-acting elements associated with stresses and hormone responses. Therefore, the expression levels of *SsVQs* under PEG, NaCl, 40 °C, ABA, SA, and MeJA treatments were further analyzed. The results indicated that the majority of the tested *SsVQ* genes were significantly upregulated by PEG, exhibiting high expression levels. Furthermore, these *SsVQ* genes contain several cis elements associated with osmotic stress or drought response. The fold change in expression was most pronounced for *SsVQ34*, suggesting that it warrants further attention in the investigation of drought resistance in willow. *SsVQ2* was consistently and significantly upregulated at a high level under both PEG and NaCl treatments. Additionally, *AtVQ15* has been confirmed to play a role in the response to high salt and osmotic stresses [11,39]. Both proteins belong to group VI are likely functionally similar. *StVQ31* is phylogenetically close to *AtVQ15* and significantly impacts osmotic and antioxidant cellular homeostasis, thereby enhancing salt tolerance [40]. Therefore, we speculate that *SsVQ2* may be involved in the response of willow to salt and osmotic stresses. The expression of *SsVQ12* and *SsVQ34* showed significant upregulation during all periods of MeJA treatment, and MeJA-responsive cis-acting elements were identified in their promoters. These genes are orthologous to *AtVQ4* and *AtVQ16*, respectively. We hypothesize that *SsVQ12* and *SsVQ34* are involved in the jasmonic acid (JA) pathway to positively regulate disease resistance in willow, as the Arabidopsis genes *AtVQ4* and *AtVQ16* have been shown to influence resistance to pathogen infection [11,41,42,43]. In addition, *SsVQ9* and *SsVQ12* exhibited significant upregulation at 40 °C, indicating their potential involvement in the response to high-temperature stress. Conversely, *SsVQ32* and *SsVQ23* demonstrated significant downregulation under ABA and SA treatments, respectively, suggesting that they may be involved in the corresponding signaling pathways.

Previous studies in Arabidopsis have demonstrated that phylogenetically closely related *VQ* genes, especially paralogous genes, often exhibit similar functions [19,20,43,44]. For instance, *VQ12* and *VQ29* negatively regulate plant basal resistance against *Botrytis cinerea*, while VQ16 and VQ23 serve as activators of *WRKY33* in the plant’s defense response to necrotrophic pathogens [20,43]. Additionally, *VQ18* and *VQ26* act antagonistically with *ABI5* to maintain appropriate levels of ABA signaling, thereby fine-tuning seed germination and early seedling establishment [19]. Consequently, we selected 13 pairs of paralogous genes from seven groups for expression level determination and correlation analysis of their expression patterns. Under six different treatments, the co-expression ratio of paralogous gene pairs is obviously higher than that of non-paralogous gene pairs (Table 2). In Arabidopsis, VQ12 and VQ29 were shown to form both homodimers and heterodimers through yeast two-hybrid and BiFC assays [20]. Furthermore, Yeast two-hybrid assays confirmed mutual interactions among Coix VQ proteins (ClVQ12, ClVQ4, and ClVQ26) [15]. Therefore, the co-expression observed in this study may be attributed to protein–protein interactions. Additionally, several paralogous pairs exhibited co-expression under various treatments. Notably, *SsVQ7* and *SsVQ17* demonstrated a consistent positive correlation across all treatment conditions; the reason for this phenomenon needs further experimental verification.

## 4. Materials and Methods

### 4.1. Identification and Analysis of the SsVQ Genes

The protein sequences of the VQ family members of *A. thaliana*, *O. sativa*, and *P. trichocarpa* were downloaded from the Phytozome v13 database (https://phytozome-next.jgi.doe.gov/ (accessed on 20 March 2025)) based on relevant literature reports [11,12,13]. The complete genome protein sequence of *S. suchowensis* was obtained from URL (https://figshare.com/articles/Willow_gene_family/9878582/1 (accessed on 20 March 2025)) [45]. Local BLASTP retrieval (E-value < 0.01) was performed to obtain candidate sequences with AtVQ protein as the seed sequence. Candidate sequences were screened by using the MEME Suite 5.5.7 online program (http://meme-suite.org/tools/meme-chip (accessed on 20 March 2025)), sequences without VQ domains were removed, and VQ members of *S. suchowensis* are named based on their chromosomal information and Sequence ID. The *SsVQ* genes were mapped onto the chromosomes based on the physical location information. Additionally, the length of sequences, isoelectric points, and molecular weights (kDa) of the *VQ* genes were calculated by the ProtParam tool (http://web.expasy.org/protparam/ (accessed on 20 March 2025)). The protein subcellular localization was predicted by the WoLF PSORT program (http://www.genscript.com/psort/wolf_psort.html (accessed on 20 March 2025)).

### 4.2. Multiple Alignment and Phylogenetic Tree Construction of the SsVQs

A multiple sequence alignment was performed using **ClustalX 2.1** to investigate the evolutionary relationships and classification of the SsVQs. The alignment results of VQ protein sequences from four plant species were selected to construct phylogenetic tree, employing 2000 bootstrap replicates through the neighbor-joining (NJ) method implemented in MEGA12 (https://www.megasoftware.net/ (accessed 20 March 2025)). Modifications to the phylogenetic tree were made using Adobe Photoshop.

### 4.3. Collinearity Analysis of the SsVQ Genes

The 36 *SsVQ* genes, except for *SsVQ37*, *SsVQ38,* and *SsVQ39,* were used for collinearity analysis. Blastn was performed to compare the *VQ* genes of *S. suchowensis*, *A. thaliana*, *O. sativa*, and *P. trichocarpa* for four genomic datasets to localize their distribution in chromosomes. Collinearity analysis was conducted using the MCScanX v1.1.11, and the results were visualized using TBtools-II v2.119 [46,47].

### 4.4. Conserved Motifs and Structure Analysis of the SsVQs

Conserved motifs of the SsVQ proteins were analyzed using the MEME website (http://meme-suite.org/tools/meme-chip (accessed on 20 March 2025)), with parameter settings of 20 motifs and a motif length range of 8–50. The 39 *SsVQ* gene sequences were uploaded to the Gene Structure Display Server website (http://gsds.gao-lab.org/ (accessed on 20 March 2025)) to analyze the gene structures, including introns, exons, and upstream/downstream untranslated sequences.

### 4.5. Identification of Cis-Elements in the Promoters of SsVQ Genes

The upstream 2000 bp DNA sequences of the *SsVQ* genes preceding the start codons were extracted as the promoter sequences. The distribution of cis elements was determined by submitting the promoter regions to PLANTCARE webtool (http://bioinformatics.psb.ugent.be/webtools/plantcare/html/ (accessed on 20 March 2025)). The cis-element analysis mainly refers to binding sites for the three classes of hormone response elements, stress-related response elements, and growth/development elements. The results were visualized using TBtools software v2.082.

### 4.6. Plant Materials, Growth Conditions, and Stress Treatments

The experiment utilized 6-week-old *S. suchowensis* seedlings in plant climate incubator under controlled conditions: 16 h light/8 h dark photo period and 25 °C day/22 °C night. Seedlings were placed in brown wide-mouth bottles and cultured with Hoagland nutrient solution, which was replaced every 7 days. To investigate the expression profiles of *SsVQ* genes under various stresses, seedlings were subjected to different treatments, including 200 mM of NaCl, 20% (*w*/*v*) PEG (polyethylene glycol 6000), 40 °C, 0.5 mmol·L^−1^ SA (salicylic acid), 0.1 mmol·L^−1^ MeJA (methyl jasmonate), and 0.1 mmol·L^−1^ ABA (abscisic acid). Leaves were taken at 0, 1, 6, 12, and 24 h after each treatment and then rapidly frozen in liquid nitrogen and stored at 80 °C for subsequent analysis.

### 4.7. RNA Extraction and qRT-PCR Analysis

Total RNA from the leaves was extracted using the Aidlab plant RNA kit (Aidlab Biotech, Beijing, China). Synthesizing cDNA used the UnionScript First-strand cDNA Synthesis Mix (Gensand, Beijing, China). The OTU (OTU-like cysteine protease family protein) gene was utilized as the internal reference for salt stress, while UBC (Ubiquitin-conjugating enzyme E2) was used for the other five treatments [48,49].

We identified 13 paralog pairs (26 genes) with sequences aligned over >300 bp and showing at least 40% identity, following the methodology by Blanc and Wolfe [50]. Subsequently, we performed qRT-PCR analysis on these 13 paralogous pairs. Primer 5 was used to design specific primers (listed in Table S3). Q-PCR was performed on CFX96TM RealTime System (Bio-Rad, Hercules, CA, USA) using TB Green Premix Ex Taq II (Tli RNaseH Plus; TaKaRa Biotechnology) in a 10 μL reaction volume with three biological and three technical replicates. The 2^−ΔΔCT^ method was used to calculate the relative expression level of each gene in this study [51]. The mean values and standard deviations (SDs) were calculated from three biological and three technical replicates. The expression data obtained from the qRT-PCR analysis were visualized as heat maps using TBtools-II v2.119.

### 4.8. Co-Expression Network Analysis

To further investigate the interaction characteristics between the expression of *SsVQ* genes within six treatments, Pearson correlation coefficients (PCC) and *p*-values were calculated using GraphPad Prism 8.0.1 software. Subsequently, gene co-expression networks were constructed using Cytoscape_v3.10.0 software [52], with PCC absolute values greater than or equal to 0.8 and *p*-values at the 0.05 significance level.

## 5. Conclusions

This study provides the first comprehensive characterization of the *VQ* gene family in *S. suchowensis*, revealing 39 *SsVQ* genes phylogenetically clustered into seven distinct groups. Evolutionary analyses indicated that whole genome duplication (WGD) and segmental duplication events drove *SsVQ* family expansion, with purifying selection playing a dominant role in their evolution. Structural conservation, including intronless architecture and conserved VQ motifs, further underscored the evolutionary stability of these genes. Notably, SsVQ19 displayed a unique hydrophilic threonine residue adjacent to the VQ amino acid residues, reflecting a characteristic observed in its homolog PtVQ27. Expression profiling under abiotic stresses (PEG, NaCl, 40 °C) and hormonal treatments (ABA, SA, MeJA) highlighted the dynamic and context-specific roles of *SsVQ* genes, with *SsVQ2*, *SsVQ9*, *SsVQ12*, *SsVQ23*, *SsVQ32*, and *SsVQ34* emerging as key candidates in stresses and hormonal-signaling pathways. Co-expression patterns among *SsVQ* genes suggested synergistic regulatory mechanisms. These findings not only enhance our understanding of the structural and functional diversification of VQ cofactors in willow but also lay a foundation for future functional studies aimed at leveraging *SsVQ* genes to improve stress resilience and productivity in woody plants through genetic engineering. Further validation of candidate genes and exploration of their molecular interactions will deepen insights into their roles in plant-environment crosstalk.

## Figures and Tables

**Table 1:** List of 39 *VQ* genes identified in *S. suchowensis* and their sequence characteristics.

Name	Sequenced ID	Protein Length (aa)	Molecular Weight (Da)	CDS Length (bp)	Exons	pI	Chromosome Location	Subcellular Location
*SsVQ1*	willow_GLEAN_10003577	128	14,077.71	387	1	4.4	chr1:1956562/1956948	Chloroplast
*SsVQ2*	willow_GLEAN_10006995	252	20,306.29	759	1	8.99	chr1:1542044/1542802	Nucleus
*SsVQ3*	willow_GLEAN_10010342	171	19,237.06	516	1	8.37	chr1:2484352/2484867	Nucleus
*SsVQ4*	willow_GLEAN_10017041	111	12,047.6	336	1	4.82	chr1:21350745/21351080	Nucleus
*SsVQ5*	willow_GLEAN_10017545	233	25,421.43	702	1	9.61	chr1:22790432/22791133	Nucleus
*SsVQ6*	willow_GLEAN_10020919	420	45,266.98	1263	1	7.06	chr2:6303101/6304363	Nucleus
*SsVQ7*	willow_GLEAN_10022200	275	30,337.21	828	2	9.46	chr2:3767449/3771058	Nucleus
*SsVQ8*	willow_GLEAN_10013497	138	15,010.84	417	1	5.76	chr3:11005186/11005602	Nucleus
*SsVQ9*	willow_GLEAN_10022134	209	22,699.94	630	1	6.28	chr3:4156214/4156843	Plasma membrane
*SsVQ10*	willow_GLEAN_10025732	320	34,874.73	963	1	10.43	chr3:5293007/5293969	Mitochondrion
*SsVQ11*	willow_GLEAN_10002711	179	19,597.23	540	1	9.48	chr4:7211068/7211607	Chloroplast
*SsVQ12*	willow_GLEAN_10017371	272	29,498.83	819	1	9.32	chr4:1785239/1786057	Nucleus
*SsVQ13*	willow_GLEAN_10005030	308	33,697.03	927	1	10.4	chr5:7911241/7912167	Cytosol
*SsVQ14*	willow_GLEAN_10006772	474	51,061.87	1425	1	7.33	chr5:2808863/2810287	Nucleus
*SsVQ15*	willow_GLEAN_10008301	209	22,596.7	630	1	6.18	chr5:1833356/1833985	Nucleus
*SsVQ16*	willow_GLEAN_10012082	447	47,594	1344	1	6.15	chr5:8238824/8240167	Nucleus
*SsVQ17*	willow_GLEAN_10015916	339	37,735.18	1020	3	9.65	chr5:10092888/10098786	Nucleus
*SsVQ18*	willow_GLEAN_10025922	176	19,198.05	531	1	9.05	chr6:11654744/11655274	Nucleus
*SsVQ19*	willow_GLEAN_10025976	105	11,418.14	318	1	9.84	chr6:12133882/12134199	Nucleus
*SsVQ20*	willow_GLEAN_10027065	134	14,522.89	405	1	4.08	chr6:1548291/1548695	Nucleus
*SsVQ21*	willow_GLEAN_10027239	252	26,918.68	759	1	9.85	chr6:293114/293872	Nucleus
*SsVQ22*	willow_GLEAN_10007056	497	53,367.44	1494	1	6.55	chr7:5496219/5497712	Nucleus
*SsVQ23*	willow_GLEAN_10012598	185	20,306.29	558	1	8.99	chr7:315837/316394	Cytosol
*SsVQ24*	willow_GLEAN_10013987	204	22,118.2	615	1	6.51	chr7:6606481/6607095	Cytosol
*SsVQ25*	willow_GLEAN_10022840	161	18,067.6	486	1	7.71	chr9:2426972/2427457	Nucleus
*SsVQ26*	willow_GLEAN_10021307	225	24,179.1	678	1	9.44	chr10:7351516/7352193	Nucleus
*SsVQ27*	willow_GLEAN_10007472	236	25,617.57	711	1	9.77	chr11:7876010/7876720	Nucleus
*SsVQ28*	willow_GLEAN_10010863	271	29,235.6	816	1	9.74	chr11:2409757/2410572	Nucleus
*SsVQ29*	willow_GLEAN_10019827	182	20,184.95	549	1	9.44	chr12:2379475/2380023	Nucleus
*SsVQ30*	willow_GLEAN_10012169	131	14,800.56	396	1	5.25	chr13:2567846/2568241	Chloroplast
*SsVQ31*	willow_GLEAN_10008946	165	18,484.41	498	1	9.01	chr14:171647/172144	Nucleus
*SsVQ32*	willow_GLEAN_10002847	186	20,439.05	561	1	7.68	chr15:4289624/4290184	Nucleus
*SsVQ33*	willow_GLEAN_10017873	222	23,897.12	669	1	4.74	chr16:9603289/9603957	Cytosol
*SsVQ34*	willow_GLEAN_10023265	134	14,840.49	405	1	5.94	chr16:1384684/1385088	Cytosol
*SsVQ35*	willow_GLEAN_10006845	178	19,382.71	537	1	7.8	chr18:380560/381096	Nucleus
*SsVQ36*	willow_GLEAN_10004237	113	12,580.16	342	1	5.14	chr19:584761/585102	Nucleus
*SsVQ37*	willow_GLEAN_10001773	256	27,397.3	771	1	5.93	scaffold01123:3188/3958	Nucleus
*SsVQ38*	willow_GLEAN_10001601	314	34,011.01	945	2	10.39	scaffold01654:1195/2291	Nucleus
*SsVQ39*	willow_GLEAN_10001403	163	17,683.21	492	1	9.17	scaffold02338:2728/3219	Nucleus

**Table 2:** Co-expression ratio statistics.

Treatments	The Co-Expressed Number of N ^a^	The Co-Expressed Ratio of N ^a^	The Co-Expressed Number of P ^b^	The Co-Expressed Ratio of P ^b^	Co-Expressed Paralogous Pairs
PEG	82	26.28	7	53.85	*SsVQ2-SsVQ37*; *SsVQ6-SsVQ16*; *SsVQ7-SsVQ17*; *SsVQ8-SsVQ34*; *SsVQ10-SsVQ38*; *SsVQ23-SsVQ31*; *SsVQ29-SsVQ32*;
NaCl	55	17.63	5	38.46	*SsVQ2-SsVQ37*; *SsVQ7-SsVQ17*; *SsVQ12-SsVQ28*; *SsVQ23-SsVQ31*; *SsVQ29-SsVQ32*;
40 °C	56	17.95	7	53.85	*SsVQ2-SsVQ37*; *SsVQ5-SsVQ27*; *SsVQ7-SsVQ17*; *SsVQ9-SsVQ33*; *SsVQ12-SsVQ28*; *SsVQ23-SsVQ31*; *SsVQ29-SsVQ32*;
ABA	35	11.22	4	30.77	*SsVQ7-SsVQ17*; *SsVQ12-SsVQ28*; *SsVQ25-SsVQ39*; *SsVQ29-SsVQ32*;
SA	41	13.14	4	30.77	*SsVQ2-SsVQ37*; *SsVQ7-SsVQ17*; *SsVQ9-SsVQ33*; *SsVQ12-SsVQ28*;
MeJA	32	10.26	4	30.77	*SsVQ7-SsVQ17*; *SsVQ9-SsVQ33*; *SsVQ10-SsVQ38*; *SsVQ23-SsVQ31*;

N ^a^ means non-paralogous gene pairs; P ^b^ means paralogue pairs; The number of gene pairs = 26 × (26−1)/2 = 325.

## Data Availability

Data are contained within the article.

## Supplementary Materials

The following supporting information can be downloaded at: https://www.mdpi.com/article/10.3390/plants14101431/s1, Figure S1: Expression analysis of *SsVQ* genes under six treatments by qRT-PCR; Table S1: Ka/Ks ratios of collinearity gene pairs; Table S2: Detailed information for the 20 motifs in the SsVQ proteins of *Salix suchowensis*; Table S3: List of primer sequences used for qRT-PCR analysis of the *SsVQ* genes from *Salix suchowensis*.
