# Supplementary figures and images for: Genome-Wide Identification, Characterization, and Expression Analysis of VQ Gene Family in Salix suchowensis Under Abiotic Stresses and Hormone Treatments
Source: Plants (Basel). 2025 May 10;14(10):1431. doi: 10.3390/plants14101431 (PMC12114880; doi:10.3390/plants14101431)

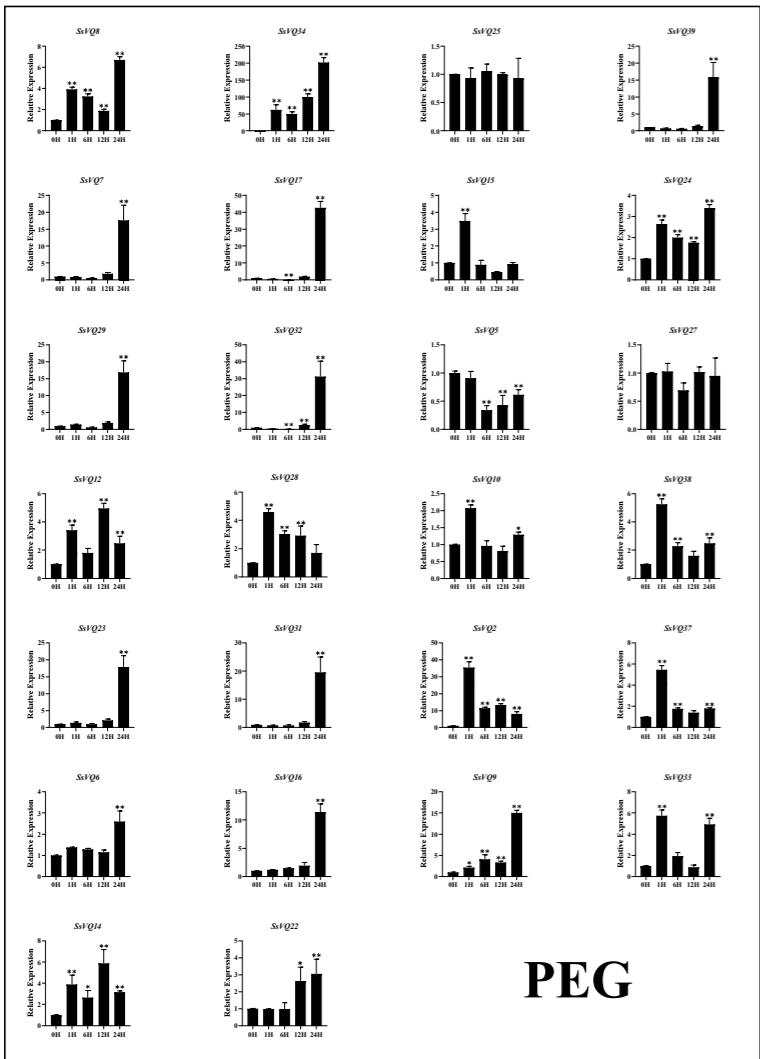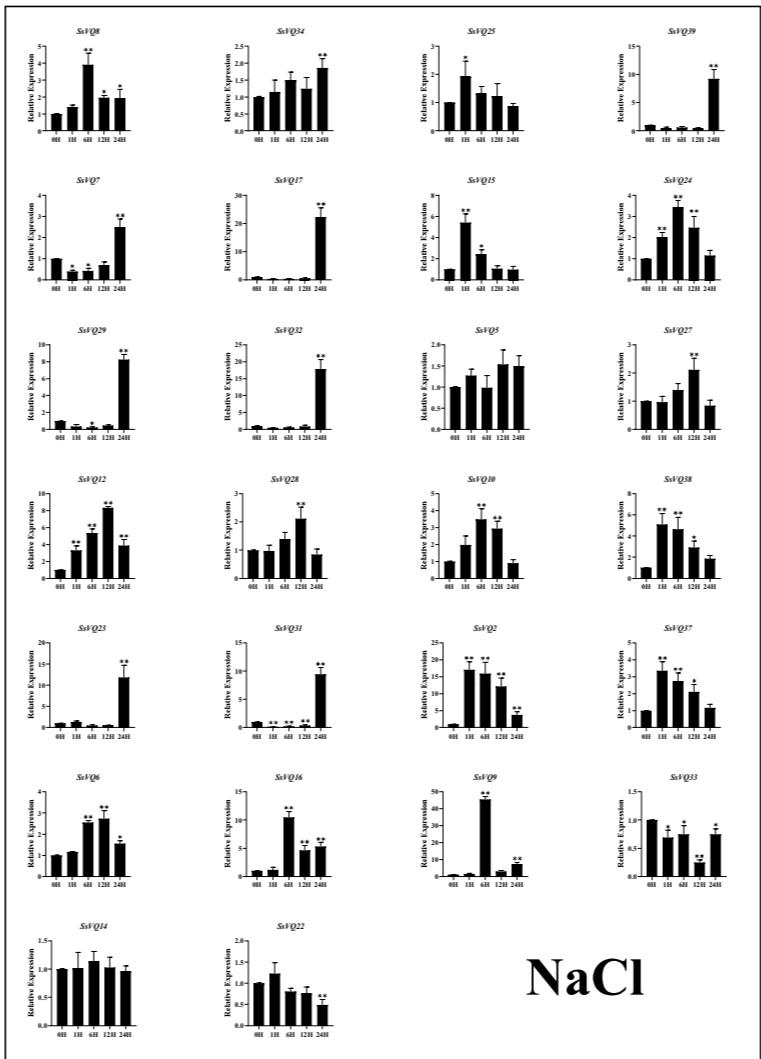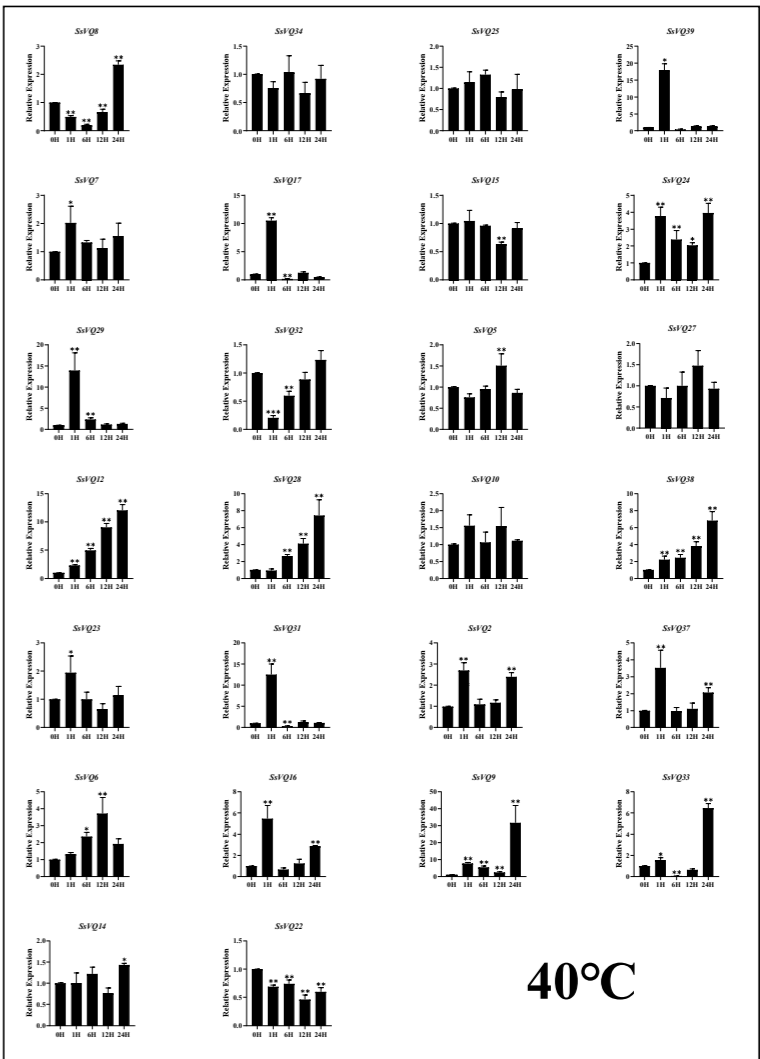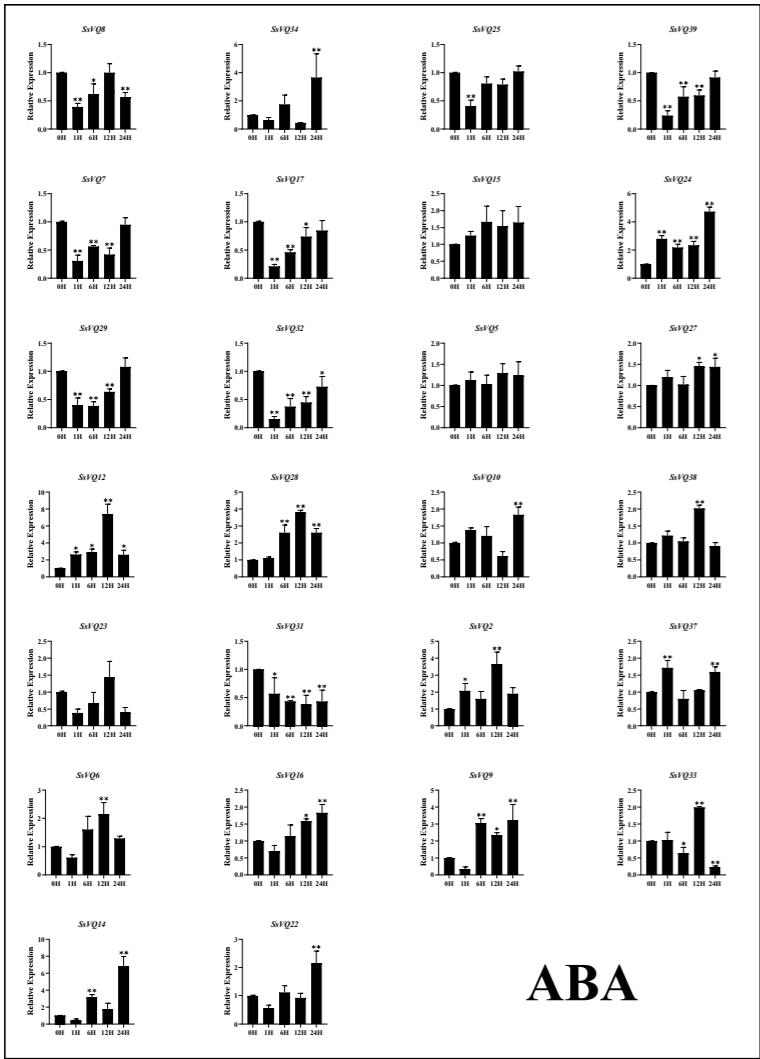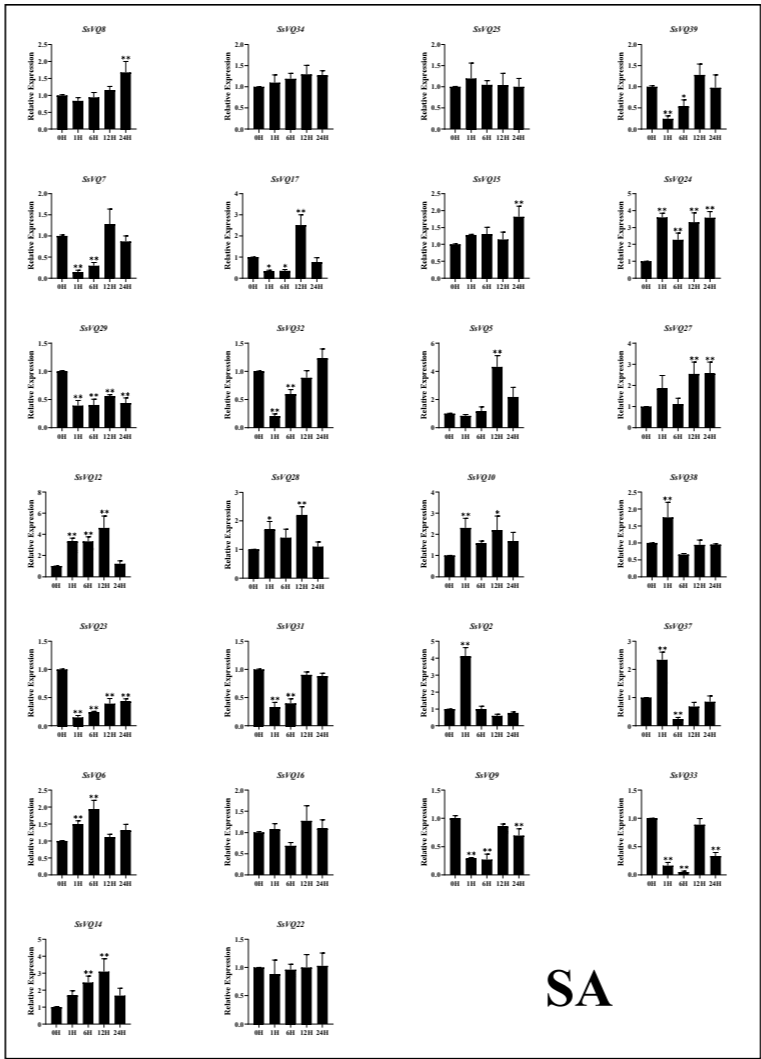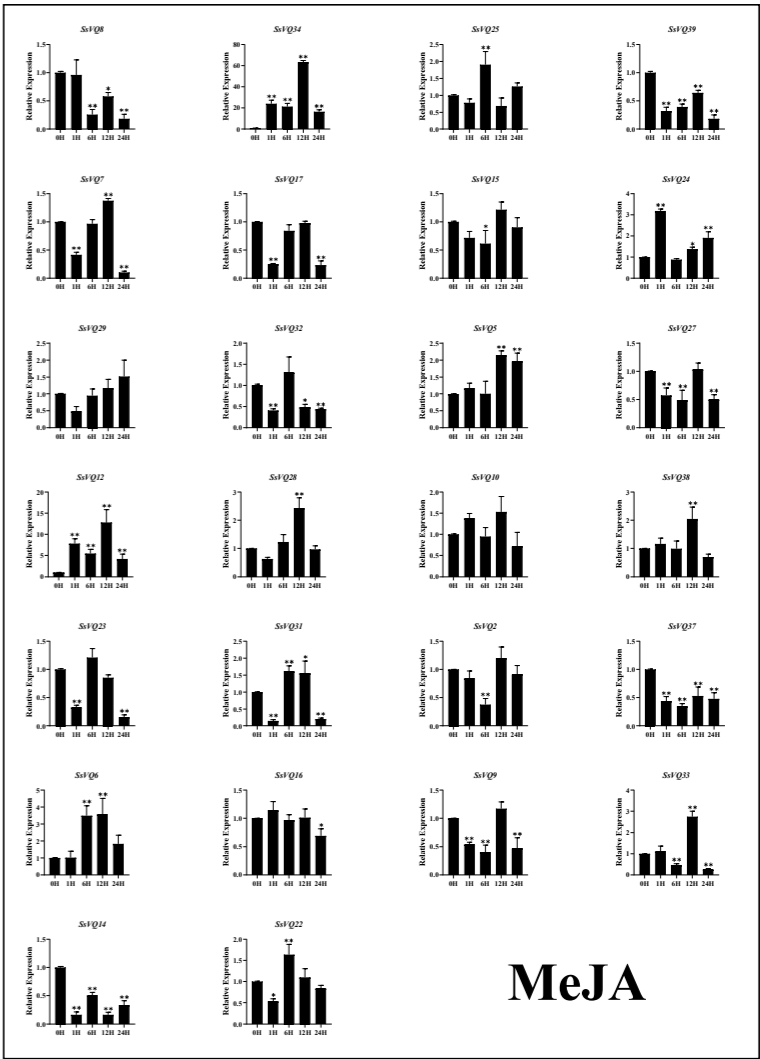

Supplement: Supplementary file 1 [file plants-14-01431-s001.zip › FIG.S1 Expression analysis of SsVQ genes under six treatments by qRT-PCR.pdf]
